# Risk Stratification in Multiple Myeloma: Putting the Pieces Together

**DOI:** 10.6004/jadpro.2016.7.3.15

**Published:** 2016-04-01

**Authors:** Angela Mayo, Craig Reeder

**Affiliations:** Mayo Clinic, Scottsdale, Arizona

Contemporary treatments have greatly extended survival in multiple myeloma (MM), and when relapse occurs, clinicians have numerous effective options at their disposal. Genetic profiling of the tumor allows for risk stratification that guides treatment selection.

"Survival is improving, thanks to new treatments," according to Angela Mayo, MS, PA-C. The proteasome inhibitors—bortezomib (Velcade), carfilzomib (Kyprolis), and ixazomib (Ninlaro, the new oral agent)—and the immunomodulating drugs (IMiDs)—thalidomide (Thalomid), lenalidomide (Revlimid), and pomalidomide (Pomalyst)—"have revolutionized treatment," she said.

"But multiple myeloma is still not a curable malignancy," she noted. "We remind our patients, this is a marathon, not a sprint, and long-term therapy will be required."

Ms. Mayo and Craig Reeder, MD, both of the Mayo Clinic in Scottsdale, Arizona, discussed how multiple myeloma is diagnosed and treated at JADPRO Live.

## MAKING THE DIAGNOSIS

Presenting signs and symptoms often include anemia and pain, especially back pain, as well as bruising/bleeding, fatigue, weight loss and frequent infections. Objective findings include anemia, lytic lesions, compression fractures, unexplained osteoporosis, pancytopenia, renal failure, and hypercalcemia, Ms. Mayo said. "Many patients, however, can be asymptomatic at diagnosis," she added.

Essential diagnostic tests include complete blood count (CBC) with differential, comprehensive metabolic panel, and serum protein electrophoresis. Clinicians will also look for serum immunoglobulins (IgA, IgG, IgM), serum free light chains (kappa FLC, lambda FLC), and beta-2 microglobulin, and will perform radiographs and a bone marrow biopsy. PET/CT can help distinguish between active disease and previous bone damage, and MRI can evaluate painful bony sites.

Monoclonal gammopathy of unknown significance (MGUS) is defined on bone marrow biopsy by < 10% monoclonal plasma cells in an asymptomatic patient. While often a precursor to MM, MGUS does not always progress. Rate of progression is 1-2% per year, and risk is cumulative, she said.

The presence of > 10% monoclonal plasma cells is diagnostic of MM, but within this category is "smoldering" MM (no end-organ damage, no need for treatment) and active disease (end-organ damage, treatment required).

The acronym CRAB indicates the diagnostic criteria for symptomatic MM:

- Calcium elevation: serum calcium ≥ 11 mg/dL
- Renal insufficiency: Creatinine clearance
  < 40 mL/min or serum creatinine
  > 2 mg/dL
- Anemia: hemoglobin < 10 g/dL or > 2 g/dL below lower limit of normal
- Bone: ≥ 1 osteolytic lesion

Without one or more of these factors, the patient does not have active MM and does not need treatment, Ms. Mayo indicated.

This classification was updated in 2014 to reflect "high-risk" smoldering MM, now classified as active disease warranting treatment: ≥ 60% plasma cells, serum light chain ratio > 100, and MRI evidence of lesions.

Staging, according to blood work, has great prognostic significance. By the International Staging System (ISS), median overall survival is 62 months for stage 1 disease, 44 months for stage II, and 29 months for stage III disease (Greipp et al., 2005).

## TREATMENT GOALS

Thanks to contemporary treatment, some patients remain disease-free for a decade or longer, however, most patients still relapse. The immediate goals are to gain rapid control of disease, reverse disease-related toxicities, and allow for stem cell collection (if patient is a transplant candidate). Long-term goals are to extend survival, prevent further organ damage, and delay relapse.

Phases of therapy are induction, stem cell collection and transplant (for most patients), maintenance, and relapse treatment.

For induction, triplet therapy has become standard, based on 5-year survival of more than 70% overall, and 80% among standard-risk patients (Reeder et al., 2014). Two common regimens are bortezomib/lenalidomide (Revlimid)/dexamethasone (VRD) and cyclophosphamide/bortezomib/dexamethasone (CyBorD).

The recent availability of highly effective agents has brought into question the need for up-front transplant. However, transplant has been shown not only to deepen responses but to improve progression-free and overall survival and it should be considered for most patients, regardless of age, according to Dr. Reeder.

Maintenance therapy is supported by studies showing both progression-free and overall survival benefits for lenalidomide/dexamethasone. While an increase in second malignancies has been observed with this regimen, many believe the benefit of staying in remission outweighs this risk, he said.

## NOVEL AGENTS AND TOXICITIES

Peripheral neuropathy is commonly associated with proteasome inhibitors. "We want to assess and intervene early," Ms. Mayo said.

Risk of neuropathy is lessened by subcutaneous or weekly administration of bortezomib, treatment breaks and reductions, and use of the newer agents in this class. Treatment of established neuropathy is less effective, but duloxetine and modification of the environment may help.

Carfilzomib (Kyprolis) is associated with less neuropathy, however, approximately half of patients will experience fatigue, anemia, and/or nausea, and about one-third will have dyspnea, thrombocytopenia and pyrexia. Dexamethasone 4 mg may help prevent flu-like reactions, Ms. Mayo said.

Advanced practitioners should be alert for worsening heart failure, cardiac events, ischemia and tumor lysis syndrome. They should also watch fluid balance and control hypertension in patients on carfilzomib.

Patients receiving any proteasome inhibitor should receive antiviral prophylaxis to avoid shingles. To manage nausea, patients can be sent home with an antiemetic.

IMiDs carry a risk for thrombosis, which can be lessened with low-dose aspirin, and a risk for fetal toxicity, therefore, pregnancy should be avoided. Neuropathy and cytopenias can also occur, as well as diarrhea, muscle cramps and fatigue. CBC should be checked regularly and patients should know the signs of a blood clot.

Lenalidomide, however, is generally well tolerated, she said, and patients can be maintained for years on this drug with a decent quality of life. Pomalidomide (Pomalyst), which was approved in 2013 for relapsed/refractory disease, is also usually well tolerated but carries the same black box warnings as the other IMiDs.

## IMPORTANCE OF RISK STRATIFICATION

"We have learned that MM is not one disease, but many," said Dr. Reeder. "The development of FISH (fluorescence in situ hybridization), which can identify mutations and extra chromosomes in plasma cells that can be high-risk features, has changed the landscape of management. We want to find high-risk patients and treat them appropriately."

Dr. Reeder summarized the most common abnormal cytogenetics and their risk stratification.

The cytogenetic classification encompasses two main groups: hyperdiploid, which generally confer a good prognosis, and non-hyperdiploid, which tend to confer worse outcomes; these are tumors with high-risk translocations (t) or fewer than the normal number of chromosomes, such as t(11;14), t(4:14), t(14:160), and deletion 17p (del(17p)). Progression-free survival is particularly poor for patients with del(17p) and t(4:14). Cytogenetics intersect with stage to impact prognosis for better or worse, he said.

"Patients with high-risk cytogenetics do worse, no matter what treatment is given, even modern three-drug regimens," he indicated. To optimize outcomes, they should receive a proteasome inhibitor.

Other factors that affect prognosis are high plasma cell labeling index, presence of plasma cell leukemia, high lactate dehydrogenase, elevated beta-2 microglobulin, low serum albumin, and failure to respond to a novel agent.

## RISK-BASED MANAGEMENT

Mayo Clinic researchers and clinicians have developed the mSMART tool—Mayo Stratification for Myeloma and Risk-Adapted Therapy—based on genetics and other prognostic factors. This is available online at www.mSMART.org and can help individualize treatment.

Dr. Reeder described the various options for treatment of transplant-eligible patients by mSMART (see Figure). Patients not going to transplant often receive the same regimens, but usually for longer duration of treatment. Options for less aggressive therapy in non-transplant patients are sequential agents (vs. all up front), CyBorD, lenalidomide/dexamethasone, and melphalan/prednisone/thalidomide.

**Figure 1:**
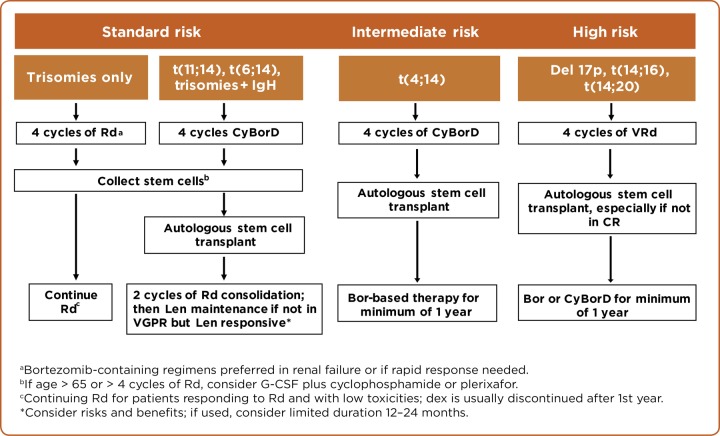
mSMART: Off-study transplant eligible. Information from Dispenzieri et al. (2007); Kumar et al. (2009); Mikhael et al. (2013).

"Relapse occurs more quickly and aggressively in high-risk patients. Close monitoring and rapid institution of therapy are critical," Dr. Reeder said. "Standard-risk patients, however, may not need rapid intervention, and can get fewer drugs, given sequentially."

Along with the proteasome inhibitors and IMiDs used in newly diagnosed patients, new options for relapse include the histone deacetylase (HDAC) inhibitor panobinostat (Farydak) and two monoclonal antibodies, daratumumab (Darzalex) and elotuzumab (Empliciti). Other agents in these classes are in clinical trials, as are numerous inhibitors of other pathways and monoclonal antibodies targeting the programmed death protein and its ligand (PD-1/PD-L1).
